# Meteorological Journal

**Published:** 1824-07

**Authors:** 


					[ 94 ]
METEOROLOGICAL JOURNAL,
From May 20, to June 19, 1824.
By Messrs. William Harris and Co. 50, Holboru, London.
May
20
21
24
25
26
27
28
29
30
31
Jun.
1
2
3
4
5
6
7
8
9
10
11 O
12
13
14
15
16
17
18
19
Kain
gauge
265
.18
?5
288
.60
.70
.25
THERM.
29.54
29.73
29.80
29.76
29.76
30.00
30.22
30.40
30.37
30.14
29.70
29.70
29.97
30.17
30.18
30.18
30.07
30.03
30.00
29.96
29.86
29.75
29.94
30.00
29.85
29.37
29.10
29.40
29.60
29.87
29.60
29.60
29.77
29.75
29.75
29.84
30.07
30.32
30.42
30.24
29.93
29.66
29.80
30.10
30.12
30.15
30.10
30.00
30.00
29.95
29.90
29.74
29.84
30.00
30.00
29.70
29.22
'.'9.27
29.46
29.80
29.84
29.32
1>E
LUC'S
HYG.
9 A.M
NW
FNE
NE
N
NW
ENE
NE
W
S
ESE
ESE
VV
N
NE
ENE
ENE
NE
NE
E
E
E
NE
ENE
NE
SW
ssw
SE
ESE
NE
NE
S
10p.m.
NNW
SE
SE
NW
E
N
SSW
E
S
E
wsw
NW
NE
?
NE
ENE
NE
E
NE
E
E
NE
NE
NE
SW
S
s
NNE
NE
W
SSW
ATMOSPHERIC
VARIATIONS.
9 A..11
Fine
Cloud.
Kain
Fair
Fine
Fail-
Cloud.
Fine
Cloud.
Fine
Cloud.
Kain
Cloud.
Fair
Rain
Fine
Kain
Fine
Kain
2 P.M.
Fine
Slio'ry
Fine
Kain
Fair
Fine
Ovcrc.
Slio'ry
Fair
Fine
Cloud.
Fine
Overc.
Fine
Rain
Fair
F air
Fair
Overc.
Fair
Fine
liain
Rain
Fine
Cloud.
Cloud.
Fine
The quantity of rain fallen in the month of May,
was 2 inches and 08. tOOths.

				

